# Haptoglobin, alpha‐thalassaemia and glucose‐6‐phosphate dehydrogenase polymorphisms and risk of abnormal transcranial Doppler among patients with sickle cell anaemia in Tanzania

**DOI:** 10.1111/bjh.12791

**Published:** 2014-02-21

**Authors:** Sharon E. Cox, Julie Makani, Deogratias Soka, Veline S. L'Esperence, Edward Kija, Paula Dominguez‐Salas, Charles R. J. Newton, Anthony A. Birch, Andrew M. Prentice, Fenella J. Kirkham

**Affiliations:** ^1^ MRC International Nutrition GroupLondon School of Hygiene & Tropical MedicineLondonUK; ^2^ Muhimbili Wellcome ProgrammeMuhimbili University of Health & Allied SciencesDar es SalaamTanzania; ^3^ Nuffield Department of Clinical MedicineUniversity of OxfordOxfordUK; ^4^ Faculty of MedicineUniversity of SouthamptonSouthamptonUK; ^5^ Department of PsychiatryUniversity of OxfordOxfordUK; ^6^ Kenya Medical Research Institute (KEMRI) Centre for Geographic Medicine Research (Coast)KilifiKenya; ^7^ Neurological Physics GroupDepartment of Medical Physics and BioengineeringUniversity Hospital Southampton NHS Foundation TrustSouthamptonUK; ^8^ Institute of Child HealthUniversity College LondonLondonUK

**Keywords:** sickle cell disease, Africa, children, cerebral blood flow velocity

## Abstract

Transcranial Doppler ultrasonography measures cerebral blood flow velocity (CBF*v*) of basal intracranial vessels and is used clinically to detect stroke risk in children with sickle cell anaemia (SCA). Co‐inheritance in SCA of alpha‐thalassaemia and glucose‐6‐phosphate dehydrogenase (*G6PD*) polymorphisms is reported to associate with high CBF*v* and/or risk of stroke. The effect of a common functional polymorphism of haptoglobin (*HP*) is unknown. We investigated the effect of co‐inheritance of these polymorphisms on CBF*v* in 601 stroke‐free Tanzanian SCA patients aged <24 years. Homozygosity for alpha‐thalassaemia 3·7 deletion was significantly associated with reduced mean CBF*v* compared to wild‐type (β‐coefficient −16·1 cm/s, *P* = 0·002) adjusted for age and survey year. Inheritance of 1 or 2 alpha‐thalassaemia deletions was associated with decreased risk of abnormally high CBFv, compared to published data from Kenyan healthy control children (Relative risk ratio [RRR] = 0·53 [95% confidence interval (CI):0·35–0·8] & RRR = 0·43 [95% CI:0·23–0·78]), and reduced risk of abnormally low CBFv for 1 deletion only (RRR = 0·38 [95% CI:0·17–0·83]). No effects were observed for *G6PD* or *HP* polymorphisms. This is the first report of the effects of co‐inheritance of common polymorphisms, including the *HP* polymorphism, on CBF*v* in SCA patients resident in Africa and confirms the importance of alpha‐thalassaemia in reducing risk of abnormal CBFv.

Although sickle cell anaemia (SCA) is a monogenic disorder caused by the homozygous inheritance of sickle haemoglobin (HbS) resulting in haemolytic anaemia, there is a wide variation in the severity and pattern of morbidities (Beutler, 2001). The contributions of other co‐inherited genetic variants on risk of severe outcomes such as stroke are under investigation (Sebastiani *et al*, 2005; Flanagan *et al*, 2011). Stroke is reported to occur in 11% of patients aged <20 years (Ohene‐Frempong *et al*, 1998; Pandey & Gorelick, 2005). Transcranial Doppler ultrasonography (TCD) is a well‐established measure of cerebral blood flow velocity (CBF*v*) of basal intracranial vessels and abnormal CBF*v* may be secondary to increased cerebral blood flow or to decrease in diameter of the vessel secondary to vasospasm or stenosis. The technique is used clinically to detect SCA patients with a high risk of stroke (Adams *et al*, 1992) with velocities of >200 cm/s and >170 cm/s predicting 40% and 7% risk of stroke over the subsequent 3 years without treatment (Adams *et al*, 1992). The risk is substantially reduced by chronic blood transfusion (Adams *et al*, 1998). Elevated CBFv is also associated with increased risk of other neurological complications, including seizures (Prengler *et al*, 2005), neurocognitive deficits (Kral *et al*, 2003; Strouse *et al*, 2006) and silent infarcts (Pegelow *et al*, 2001). Low velocities may also be seen in patients with cerebrovascular disease, consistent with proximal vascular stenosis (Kirkham *et al*, 1986) or occlusion (Lee *et al*, 2004) and appears to be associated with cerebrovascular accidents (Buchanan *et al*, 2012).

Haptoglobin (HP) is an acute phase protein that removes free haemoglobin (Hb) from the circulation after haemolysis or tissue damage, and is therefore an important anti‐oxidant. HP is encoded by the co‐dominant alleles, HP*1 and HP*2, resulting in three distinct phenotypes HP11, HP12 and HP22. Plasma concentrations of HP, and its binding affinities for free Hb, vary by phenotype, with HP22 reported to have reduced overall Hb affinity (Langlois & Delanghe, 1996), less efficient antioxidative capacity, (Langlois & Delanghe, 1996; Melamed‐Frank *et al*, 2001) and reduced clearance rates of HP2‐Hb complexes by macrophages (Asleh *et al*, 2003). This is suggested to result in increased nitric oxide scavenging by HP2‐Hb complexes (Azarov *et al*, 2008), with reduced downstream anti‐inflammatory signalling after macrophage endocytosis of HP2‐Hb complexes (Landis *et al*, 2013), weaker inhibition of prostaglandin synthesis and a stronger angiogenic effect (Cid *et al*, 1993). There is strong evidence that the HP22 phenotype is associated with increased risk of stroke and cardiovascular events in diabetic patients (Vardi *et al*, 2012). Furthermore, several reports demonstrate an association between the HP*2allele with vasospasm after subarachnoid haemorrhage; as determined by high CBFv (Borsody *et al*, 2006), angiographically (Ohnishi *et al*, 2013), and in a mouse model (Chaichana *et al*, 2007). Vasospasm after sub‐arachnoid haemorrhage is thought to result from inflammation, oxidative stress, nitric oxide scavenging, leucocyte migration and endothelial‐leucocyte interactions (Chaichana *et al*, 2010), all from extracellular Hb. In HP22 mice with sub‐arachnoid haemorrhage, treatment with an anti‐oxidant (Froehler *et al*, 2010) or controlled nitric oxide (Momin *et al*, 2009) reduces vasospasm, which is supportive evidence that these mechanisms are important. The HP*2allele is also associated with increased severity and adverse outcomes in various infections including human immunodeficiency virus (HIV and malaria (Cox *et al*, 2007; McDermid & Prentice, 2006). Effects of HP phenotype on cerebrovascular outcomes in non‐diabetic patients, e.g. those with SCA, have not been reported.

Although the co‐inheritance of α‐thalassaemia deletions and glucose‐6‐phosphate dehydrogenase (G6PD) deficiency have been previously investigated as disease modifiers in SCA, specifically affecting risk of elevated CBFv and/or stroke, these have not been investigated in Africa. The co‐inheritance of alpha‐thalassaemia in SCA modifies red cell indices (Embury *et al*, 1984; Stevens *et al*, 1986; Kulozik *et al*, 1988) and red cell rheology (Serjeant *et al*, 1983), and, in some reports, increases total Hb (Embury *et al*, 1982). Alpha‐thalassaemia is consistently associated with a decreased risk of increased CBFv and/or stroke in SCA (Adams *et al*, 1994; Hsu *et al*, 2003; Bernaudin *et al*, 2008; Belisario *et al*, 2010; Flanagan *et al*, 2011). G6PD deficiency results in decreased capacity to reduce oxidized glutathione via NADPH and thus reduced ability of red cells to counteract oxidant stress (Mason *et al*, 2007). G6PD deficiency (A‐genotype) in SCA is associated with lower Hb but not increased haemolysis (Nouraie *et al*, 2010). Reports of the effects of co‐inheritance of G6PD deficiency with CBFv, vasculopathy on magnetic resonance angiography and/or stroke risk are contradictory (Bernaudin *et al*, 2008, 2011; Rees *et al*, 2009; Miller *et al*, 2011; Thangarajh *et al*, 2012) and there are no data from patients resident in Africa.

We hypothesized that the co‐inheritance of the HP*2allele in children with SCA would increase the risk of high CBFv and furthermore, that there may be epistatic effects between the three unrelated polymorphisms under investigation such that an effect may be greatest in, or limited to, children with the HP*2allele who also co‐inherited G6PD deficiency and did not have alpha thalassaemia.

## Methods

Ethical approval was granted by the Muhimbili University of Health and Allied Sciences, Tanzania (MU/RP/AEC/VOL XI/33), and the London School of Hygiene and Tropical Medicine, UK (reference 5158) Review Boards. All participants, parents or guardians gave written informed consent (in Kiswahili) for participation at enrolment into the SCA clinical cohort at Muhimbili National Hospital (MNH).

### Study population and Transcranial Doppler examination

Participants aged <24 years were enrolled in the Muhimbili Sickle Cohort at Muhimbili National Hospital, Tanzania and had a TCD examination at one or more of three cross‐sectional surveys, conducted in 2004/05, 2009 and 2010. Patients who received blood transfusion within the previous two months, symptoms of sickle crisis within the previous 2 weeks or a previous history of stroke were excluded. TCD examinations were performed according to the STOP (Stroke prevention in sickle cell disease) protocol (Nichols *et al*, 2001) using the Companion II (Nicolet, Warwick, UK). CBFv was determined as the highest time averaged maximum velocity in the distal internal carotid artery and middle cerebral artery on either side. CBFv was classified using data from 115 Kenyan non‐SCA control children (Newton *et al*, 1996) as low (<43 or <39·5 cm/s) for the left and right middle cerebral artery (LMCA/RMCA), high (>141 or >143·5 cm/s LMCA/RMCA) or normal. CBFv were also classified as conditional (170–199 cm/s) or abnormal (>200 cm/s) based on previous criteria (Adams *et al*, 1990; Newton *et al*, 1996). In addition, the difference between the maximum velocities in the ipsilateral to contralateral MCA was calculated; this was previously shown to predict vasospasm in patients after sub‐arachnoid haemorrhage (Nakae *et al*, 2011). The difference between the two sides was then calculated as a percentage of the lower velocity and categorized to indicate the degree of asymmetry between the left and right MCA as: no asymmetry – values less than 40%, degree 1; values 40–75%, degree 2; 75–180%, degree 3; and, >180%, degree 4. These cut‐off points are currently used clinically to follow vasospasm after subarachnoid haemorrhage at University Hospital Southampton.

### Laboratory procedures

Sickle status was diagnosed and quantification of Hb fractions was performed by high performance liquid chromatography (HPLC) using the *β*‐thalassaemia Short Program on the Variant I analyser (BioRad, Hercules, CA, USA). Full blood counts were performed using an automated cell counter (Pentra 60, Horiba ABX, Kyoto, Japan) within 7 d of the TCD study. Genomic DNA was isolated from peripheral blood leucocytes using Nucleon kits (BACCII). *HP* functional variants (alleles HP*1 and HP*2) were genotyped by allele‐specific polymerase chain reaction (PCR) adapted from published techniques (Koch *et al*, 2002; Cox *et al*, 2007). Individuals were genotyped for the 3·7 alpha‐thalassaemia deletion using a PCR‐based method and agarose gel visualization as per published methods (Williams *et al*, 2005). The 202‐ and 376‐single nucleotide polymorphisms (SNPs) (rs1050828 [G‐202A] & rs1050829 [A‐376G]), the combined inheritance of which results in the A‐ phenotype of glucose 6‐phosphate dehydrogenase (G6PD) deficiency, and HbS (rs334) were determined using multiplex Sequenom^®^ MassARRAY^®^ (Sequenom^®^, Hamburg, Germany).

### Statistical analysis and sample size

In the current analysis one observation per subject was selected according to the observation that recorded the highest CBFv, assessed separately in the l‐ and RMCA. Statistical analyses were performed with stata ic software (version 12.0, StataCorp, College Station, TX, USA). Categorical variables were constructed for the CBFv values, using the Kenyan and STOP cut‐off points as described above. We used two approaches to analyse the CBFv outcome. In the first we used multiple linear regression to explore predictors of CBFv as a continuous variable, whilst excluding those with absent signal. We determined that, using this approach, 90 children per group (HP22 vs. HP11) were required to detect a difference in mean CBFv equivalent to 0·5 × standard deviation (SD) of mean CBFv, based on data from similar aged Kenyan children with SCA (Makani *et al*, 2009) with 90% power and two‐tailed significance at 5%. In the second approach, we used multinomial logistic regression to explore predictors of CBFv as a categorical variable representing potential cardiovascular disease (CVD) (allowing simultaneous investigation of factors affecting risk of low *vs*. normal and high *vs*. low CBFv, presenting the results as relative risk ratios (RRRs) with 95% confidence interval (CI). A *P*‐value <0·05 was considered statistically significant.

## Results

### Characteristics of the study population

A total of 601 homozygous SCA patients were included. The demographic, laboratory and CBFv characteristics of the patients are summarized in Table 1. The age of the patients ranged from 0·6 to 22·6 years, with 12% aged <5 years and 2·5% aged >16 years. The majority of subjects (51%) were included from the largest survey in 2004/2005. There was no evidence of a difference between the surveys for sex, genotype or Hb concentrations. However, mean age and HbF% were significantly lower in the 2004/05 survey. All the participants had genotype results for at least one of the three polymorphisms under investigation, with 385 having complete data for the three genes being investigated.

**Table 1:** Patient characteristics.

Demographic variables	Summary statistic	Observations (*N*)
Age [years], mean (SD)	9·76 (3·86)	601
Males, *n* (%)	325 (54·08)	601
Survey, *n* (%)		
2004/05	305 (50·75)	601
2009	224 (37·27)	
2010	72 (11·98)	
Laboratory variables^a^		
Haemoglobin [g/l], mean (SD)	74·0 (11·2)	583
Fetal haemoglobin [%], mean (SD)	5·23 (3·84)	533
CBFv		
MCA maximum velocity, left or right, *n* (%)		
Kenyan non‐SCA criteria^b^		601
Normal	387 (64·39)	
Low [≤43 cm/s or <39·5 cm/s]	44 (7·32)	
High [>141 cm/s or >143·5 cm/s]	170 (28·29)	
STOP criteria		
Normal	534 (88·85)	
Conditional >170 < 200 cm/s	25 (4·16)	
Abnormal ≥200 cm/s	42 (6·99)	
Asymmetry (Vasospasm)^c^ *n* (%)		
No asymmetry	317 (54·75)	579
Mild asymmetry	104 (17·96)	
Moderate asymmetry	93 (16·06)	
Severe asymmetry	65 (11·23)	

SD, standard deviation; CBFv, cerebral blood flow velocity; SCA, sickle cell anaemia; STOP, stroke prevention in sickle cell disease; MCA: middle cerebral artery.

a All laboratory values were assessed within 7 d of the Transcranial Doppler (TCD) measurement, except HbF which if measured under the age of 60 months and not within 7 d of the TCD was not included.

b Based on values from healthy Kenyan children (mean ± 2 SD)(Newton *et al*, 1996).

c Degree of asymmetry is difference in the maximum velocities in the left MCA and right MCA, calculated as a percentage of the lower velocity. Mild = 40–75%, moderate ‐ 75–180%, severe >180%.

Mean CBFv was 131 cm/s (SD = 42). The proportion of non‐detectable CBFv in the LMCA compared to the RMCA was significantly different (*P* < 0·006) at 5% for the LMCA and 8% for the RMCA. However, there was no significant difference in mean CBFv between the left and right MCA (120 cm/s [SD 42, *N* = 571] vs. 119 cm/s [SD 42, *N* = 552]).

The genotype prevalence data are summarized in Table 2. The prevalence of the inherited combinations of the three genes in the 385 participants with a complete dataset is available as supplementary material in Table SI.

**Table 2:** Genotype prevalence and scores assigned to each genotype.

Gene	Homozygote WT	Heterozygote	Homo/hemizygote mutant
*G6PD* A‐Phenotype (202 & 376) *N* = 583	BB [75·30%]	A‐B [11·32%]	A‐Z or A‐A‐[13·38%]
3·7 Alpha‐thalassaemia deletion *N* = 568	αα/αα [43·66%]	α‐/αα [40·67%]	α‐/α‐ [15·67%]
*HP N* = 422	HP11 [23·46%]	HP12 [56·64%]	HP22 [19·91%]

### Factors associated with TCD outcomes

In the first approach we used linear regression to investigate possible associations with CBFv for age, sex, Hb and HbF%, as well as the three genes under investigation, including all measureable CBFv observations. The results are summarized in Table 3. Strong inverse associations with CBFv were apparent for age and Hb. Initial analyses of the genotypes were adjusted for age and year of survey. Hb concentration was not immediately adjusted for, as all of the genotypes under investigation may potentially act, at least in part, through effects on Hb. Inheritance of two copies of the 3·7 α‐thalassaemia deletion, compared to no copies, was significantly associated with decreased mean CBFv. If Hb was adjusted for, no effect of α‐thalassaemia was observed, suggesting the effect is mediated via Hb. There were no apparent effects of G6PD status or *HP* genotype, even when when limited to those with gene combinations hypothesized as the ‘worst’ and ‘best combinations ([HP22/A‐A‐or A‐Z, and/or, αα/αα] vs. [HP11/BB/‐α/‐α]).

**Table 3:** Associations between demographic, laboratory variables and genetic variants with CBFv as a continuous measurement^a^.

Predictors	β‐Coefficient (95% CI)	*P*‐value	Observations (*n*)
Age (years)^b^	−2·28 (−3·21 to −1·35)	<0·0001	525
Sex (male vs female)^b^	3·512 (−3·56–10·58)	0·329	525
Haemoglobin (g/l)^b^	−4·60 (−7·91 to−1·29)	0·007	525
Fetal haemoglobin (%)^b^	−0·197 (−1·11–0·72)	0·672	525
Haptoglobin HP11^c^			
HP12	0·28 (−9·61–10·17)	0·956	410
HP22	−2·994 (−15·32–9·33)	0·633	
α‐Thalassaemia normal^c^			
1 Deletion	−6·32 (−13·78–1·14)	0·097	549
2 Deletions	−16·14 (−26·271 to −6·0135)	0·002	
G6PD Phenotype normal^c^			
Mild (A‐B)	−8·80 (−19·44–1·84)	0·105	564
Affected (A‐Z or A‐A‐)	−1·81 (−11·83–8·21)	0·723	

a Only velocity values >0 cm/s included for multiple linear regression.

b Results for multivariable regression including the marked variables.

c Adjusted for age and survey year CBFv, cerebral blood flow velocity; 95% CI, 95% confidence interval.

In the second approach we explored associations using multinomial logistic regression, in which CBFv was categorized as low, normal or high (Table 4). The only genotype with evidence of an association was the 3·7 α‐thalassaemia deletion, the inheritance of which was associated with a significantly decreased risk of having an abnormally high or low CBFv.

**Table 4:** Relative risk ratios of genetic variants on CBFv categories defined as low or high compared to normal using data from Kenyan healthy non‐SCA child population adjusted for age and year of survey.

Genotypes	Relative risk ratio (95% confidence interval)^a^		*N*
	(CBF*v* Low vs. normal)	(CBF*v* High vs. normal)	
Haptoglobin, HP11			
HP12	0·90 (0·33–2·47)	0·90 (0·53–1·51)	422
HP22	1·01 (0·30–3·38)	0·85 (0·44–1·66)	
α‐Thalassaemia, normal			
1 Deletion	0·38 (0·17–0·83)^b^	0·53 (0·35–0·80)^b^	568
2 Deletions	0·95 (0·41–2·20)	0·43 (0·23–0·78)^b^	
G6PD Normal phenotype			
Mild	0·49 (0·14–1·65)	0·62 (0·32–1·17)	583
Affected	0·41 (1·12–1·37)	0·78 (0·44–1·39)	

a Low CBFv corresponds to values <43 in LMCA or <39·5 in RMCA, including values of 0; High CBFv corresponds to values >141·1 in LMCA or >143·6 in RMCA), compared to normal (CBFv: 43–141 cm/s in LMCA or 39·6–143·5 cm/s in RMCA)(Newton *et al*, 1996).

b *P*‐value <0·05.

RRR: Relative risk ratio; CBFv, cerebral blood flow velocity; SCA, sickle cell anaemia; RMCA, right middle cerebral artery; LMCA, left middle cerebral artery.

We also investigated genotype associations with abnormal CBFv according to the STOP classifications (normal <170 cm/s, conditional 10–199 cm/s and abnormal ≥200 cm/s) (Adams *et al*, 1998) but no significant associations were observed.

Finally we also investigated possible associations with the degree of asymmetry in CBFv, classified as normal, mild, moderate or severe. No consistent effects were observed for any of the variables tested (Table 5).

**Table 5:** Relative risk ratios of genetic variants on category of vascular asymmetry as an indicator of vasospasm adjusted for age and year of survey.

Genotypes	Relative risk ratio (95% confidence interval)^a^			*N*
	Mild *vs*. normal	Moderate *vs*. normal	Severe *vs*. normal	
Age^b^	0·08 (0·02–0·14)[Link]	−0·01 (−0·08–0·05)	0·03 (−0·04–0·11)	525
Sex^b^	0·01 (−0·48–0·50)	−0·08 (−0·58–0·42)	0·04 (−0·54–0·62)	
Fetal Haemoglobin^b^	−0·03 (−0·09–0·04)	−0·02 (−0·09–0·04)	−0·03 (−0·11–0·05)	
Haemoglobin^b^	0·10 (−0·13–0·34)	−0·27 (−0·50 to −0·03)[Link]	−0·08 (−0·35–0·20)	
Haptoglobin, HP11				
HP12	1·07 (0·58–1·98)	1·12 (0·56–2·21)	1·25 (0·56–2·79)	410
HP22	0·86 (0·39–1·90)	1·21 (0·53–2·75)	1·12 (0·41–3·03)	
α‐Thalassaemia, normal				
1 Deletion	1·69 (1·01–2·84)[Link]	0·98 (0·58–1·64)	0·94 (0·52–1·70)	549
2 Deletions	1·66 (0·86–3·23)	0·99 (0·49–1·98)	0·52 (0·20–1·35)	
G6PD Normal phenotype				
Mild (A‐B)	0·98 (0·48–1·98)	0·94 (0·44–1·99)	0·95 (0·39–2·31)	564
Affected (A‐Z or A‐A‐)	0·77 (0·38–1·58)	1·11 (0·57–2·16)	0·85 (0·35–2·03)	

a Degree of asymmerty is difference in the maximum velocities in LMCA and RMCA calculated as a percentage of the lower velocity. Mild = 40–75%, moderate −75–180%, severe >180%.

b Results for multivariable regression including the marked variables.

*P*‐value <0·05; Adjusted for survey year.

## Discussion

This is the largest study to date to assess the effects of the disease modifying and commonly co‐inherited polymorphisms of alpha‐thalassaemia and *G6PD* on prospectively measured CBFv in SCA, and the first in children and adolescents resident in Africa. In addition, this is the first study to assess the effect of co‐inheritance of *HP* polymorphisms on a clinical end‐point in SCA. We confirm previous reports of a reduction in mean CBFv and protection from abnormal CBFv measurements in heterozygote and homozygotes for the α‐thalassaemia 3·7 deletion. This effect was not significant when limited to abnormal, as defined by the STOP criteria. We also observed a significantly decreased risk of abnormally low CBFv in alpha thalassaemia heterozygote children. No effects of *G6PD* or *HP* polymorphisms were observed, including when assessed in children with the hypothesized ‘worst’ gene combinations compared to ‘optimal’ combination.

Mean CBFv (131 cm/s) and the prevalence of highly elevated (>200 cm/s) (7%) and conditionally elevated (170–199 cm/s) (4%) CBFv were higher in our Tanzanian patients compared to rural Kenyan children with SCA with a mean CBFv of 120 cm/s and only 3% with conditional and none with high CBFv (Makani *et al*, 2009), but still considerably lower than in other studies in which alpha‐thalassaemia and/or G6PD were also assessed. Sixteen percent of French children with sickle cell disease had CBFv ≥200 cm/s (62/373) (Bernaudin *et al*, 2008), whilst 18% (409/2334) had CBFv of 170–199 cm/s and 9% >200 cm/s (217/2324) in the baseline measurement of the STOP study in the USA (Adams *et al*, 2004). However, the proportions were similar to that reported in Brazilian patients with SCA/sickle cell disease in whom 4% (7/164) had CBFv 170–199 cm/s and 3% (5/164) had a CBFv >200 m/s (Belisario *et al*, 2010). In both the Brazilian and the French cohorts the 3·7 alpha‐thalassaemia deletion was associated with protection from conditional or high CBFv, whilst in the STOP trial cohort a similar finding was observed in a case control design (Hsu *et al*, 2003). Thus our results confirm a protective effect of the alpha‐thalassaemia 3·7 deletion, resulting in a lower mean CBFv and reducing the risk of even moderate elevations, compared to normal, and importantly, also protecting against abnormally low CBFv, also an indicator of CVD and stroke risk. However, the overall protective effect of alpha‐thalassaemia status on risk of stroke in our patients remains to be determined and may differ from other populations less dominated by the Central African Republic (CAR) haplotype. No protective effect of altha‐thalassaemia was apparent for degree of asymmetry between the ipsilateral and contralateral MCA. The effect of the alpha‐thalassaemia deletion may be mediated at least in part via increased Hb. Co‐inheritance of the 3·7 deletion is consistently and quantitatively associated with decreased mean cell volume (MCV), mean cell haemoglobin (MCH), mean cell haemoglobin concentration (MCHC) and haemolytic markers, including in this population, (Cox *et al*, 2013) and with decreased WBC (Bernaudin *et al*, 2008). HbS concentration is the primary determinant of HbS polymerization (Eaton & Hofrichter, 1987). Thus the protective effect of co‐inheritance of the 3·7 deletion has been commonly assumed to be due to decreased sickling rates (Ballas, 2001) and consequent haemolysis. However, in the analysis by Bernaudin *et al* (2008) there were independent effects on CBF*v* of the 3·7 deletion after adjustment for MCV (MCH & MCHC not analysed), white blood cell count and the haemolytic marker lactate dehydrogenase (LDH) (itself independently associated in the multivariate model). The authors suggested increased red cell deformability as a potential mechanism. In a recent, large, carefully controlled study, co‐inheritance of the 3·7 deletion in SCA was quantitatively and negatively associated with the proportion of dense dehydrated red cells (%DRBCs) measured at steady state. Increased%DRBC was, in turn, positively associated with risk of priapism, leg ulcers and renal dysfunction, of which only renal dysfunction was associated with haemolysis (Bartolucci *et al*, 2012). There is evidence to suggest that the increased rigidity of DBRCs may promote vasoconstriction via reduced shear stress‐induced ATP release as a vasodilatory signalling molecule (Wan *et al*, 2008).

It is unlikely that the lack of an effect of the HP*2allele was due to a type II error as a result of a lack of power, as evidenced by our adequate sample size of 83 HP22 and 103 HP11 compared to our sample size estimate of 90 per group to detect an effect equivalent to half a standard deviation (SD) in CBF*v*. Thus although there was a probable increased variance in our data due to different observers between survey years, a similar effect size was observed for alpha‐thalassaemia in this study (α‐/α‐ (*N* = 94) vs. αα/αα (*N* = 258), β‐coefficient −16·14 cm/s = 0·4 SD of CBFv). However, the 95% CIs of the observed effect of HP22 did just include the effect size in the sample size calculation (−15·3 cm/s vs. 0·5 × SD = 20·9). Thus, it remains a possibility that small effects of the HP*2allele exist, reducing risk of either both or the lower extremes of CBF*v*, not captured in the multinomial logistic model, which was not included in pre‐study sample size calculations. Thus despite the strong evidence for a mechanism whereby the HP22 variant could be expected to increase vascular activation and vasoconstriction in the inflammatory and haemolytic condition of SCA, our data does not support this suggestion. An explanation for this could be that HP is overwhelmed in SCA, such that phenotypic variations in its functions cease to be relevant. This scenario is supported by our observation that during well, steady‐state clinic visits, HP levels were non‐detectable in 96% of our patients (lower limit of detection = 0·04 g/l, *N* = 838) compared to 17% of non‐SCA controls and not affected by genotype (S. E. Cox, J. Makani & A. M. Prentice, unpublished data). A possible alternative explanation is that in HP22 individuals the haem‐oxygenase‐1 compensatory pathway may be more successfully up‐regulated in response to haem exposure, demonstrated to have powerful anti‐inflammatory and anti‐stasis effects (Jison *et al*, 2004; Belcher *et al*, 2006).

Conflicting reports of an effect of *G6PD* on CBFv (Bernaudin *et al*, 2008, 2011; Rees *et al*, 2009; Miller *et al*, 2011; Thangarajh *et al*, 2012) may result from variations between populations in either the phenotypic expression compared to assessed genotype or from population or methodological differences between *in‐vivo vs. in‐vitro* enzyme activity, when this has been measured directly (Johnson *et al*, 2009). There is little evidence for an association of either low enzyme activity (Miller *et al*, 2011) or genotype (Flanagan *et al*, 2011) with stroke. Decreased enzyme activity was associated with TCD abnormality in children resident in France (Bernaudin *et al*, 2008) and this effect appeared to be independent of haemolysis. However, this association was not observed in England (Rees *et al*, 2009). Genotypes rs1050828 or rs1050829 were associated with magnetic resonance angiography evidence of vasculopathy in a cohort from the US, England and France but children with TCD abnormality were excluded (Thangarajh *et al*, 2012). It is possible that *G6PD* polymorphisms play a role in the initiation or development of vasculopathy in certain environments but we have no evidence for this in our population resident in Africa and the lack of an association with stroke suggests that other risk factors may be more important.

In conclusion, despite adequate power to determine a clinically relevant effect size of the HP*2allele on CBFv, we could determine no evidence of such an effect. In a population of SCA patients resident in Africa, we confirmed the effect of alpha‐thalassaemia, but could not find an effect of *G6PD*.

## Funding

This work was supported by the Wellcome Trust UK: (SC) project grant 080025; (JM) personal fellowship 072064; strategic awards 084538 and 095009.

## Supplementary Material

**Table SI.** Proportions of subjects for each combination of the three genotypes under investigation.
