# Supplementary material for: Haptoglobin, alpha‐thalassaemia and glucose‐6‐phosphate dehydrogenase polymorphisms and risk of abnormal transcranial Doppler among patients with sickle cell anaemia in Tanzania
Source: Br J Haematol. 2014 Feb 21;165(5):699–706. doi: 10.1111/bjh.12791 (PMC4154124; doi:10.1111/bjh.12791)
Supplement: Supplementary file 1 — Table SI. Proportions of subjects for each combination of the three genotypes under investigation. [file bjh-165-699-s1.doc]

**Table SI. Proportions of subjects for each combination of the 3 genotypes under investigation**

Expressed as % of total [N=601]

Colour coding, blue to red indicates hypothesised effect of gene combinations from the most beneficial to the least.

|  |  | **G6PD A- phenotype** | | |  |  |
| --- | --- | --- | --- | --- | --- | --- |
|  |  | norm | mild | affected |  |  |
| **HP** | HP11 | 7.7 | 1.2 | 1.3 | norm | **a-thal** |
|  |  | 7.5 | 1.4 | 1.0 | 1 deletion |  |
|  |  | 3.0 | 0.4 | 0.4 | 2 deletions |  |
|  | HP12 | 17.9 | 2.7 | 3.6 | norm |  |
|  |  | 18.8 | 2.2 | 3.2 | 1 deletion |  |
|  |  | 6.5 | 0.8 | 0.5 | 2 deletions |  |
|  | HP22 | 6.1 | 1.2 | 1.7 | norm |  |
|  |  | 5.7 | 1.4 | 1.0 | 1 deletion |  |
|  |  | 2.2 | 0.1 | 0.4 | 2 deletions |  |
